# Modulating Behavior in *C*. *elegans* Using Electroshock and Antiepileptic Drugs

**DOI:** 10.1371/journal.pone.0163786

**Published:** 2016-09-26

**Authors:** Monica G. Risley, Stephanie P. Kelly, Kailiang Jia, Brock Grill, Ken Dawson-Scully

**Affiliations:** 1 Department of Biological Sciences, Florida Atlantic University, Boca Raton, Florida, United States of America; 2 Department of Neuroscience, The Scripps Research Institute, Scripps Florida, Jupiter, Florida, United States of America; INSERM U869, FRANCE

## Abstract

The microscopic nematode *Caenorhabditis elegans* has emerged as a valuable model for understanding the molecular and cellular basis of neurological disorders. The worm offers important physiological similarities to mammalian models such as conserved neuron morphology, ion channels, and neurotransmitters. While a wide-array of behavioral assays are available in *C*. *elegans*, an assay for electroshock/electroconvulsion remains absent. Here, we have developed a quantitative behavioral method to assess the locomotor response following electric shock in *C*. *elegans*. Electric shock impairs normal locomotion, and induces paralysis and muscle twitching; after a brief recovery period, shocked animals resume normal locomotion. We tested electric shock responses in loss-of-function mutants for *unc-25*, which encodes the GABA biosynthetic enzyme GAD, and *unc-49*, which encodes the GABA_A_ receptor. *unc-25* and *unc-49* mutants have decreased inhibitory GABAergic transmission to muscles, and take significantly more time to recover normal locomotion following electric shock compared to wild-type. Importantly, increased sensitivity of *unc-25* and *unc-49* mutants to electric shock is rescued by treatment with antiepileptic drugs, such as retigabine. Additionally, we show that pentylenetetrazol (PTZ), a GABA_A_ receptor antagonist and proconvulsant in mammalian and *C*. *elegans* seizure models, increases susceptibility of worms to electric shock.

## Introduction

*Caenorhabditis elegans* has begun to emerge as a powerful *in vivo* model for research on neurological conditions including neurodegenerative diseases and neurodevelopmental disorders [1–7]. In comparison to mammals, *C*. *elegans* possesses a relatively simple nervous system, but has important conserved features of nervous system function at the level of ion channels and neurotransmitters. Of particular relevance to this study is the conservation of gamma-aminobutyric acid (GABA) and GABA receptors in inhibitory neurotransmission, and acetylcholine (ACh) and acetylcholine receptors in excitatory neurotransmission [8–11]. In addition to physiological similarities, *C*. *elegans* has a fully mapped connectome, is small and inexpensive to maintain and preserve, has a short three-day generation time, and is conducive for large-scale screening [12–17].

In the past, studies investigating *C*. *elegans* as a convulsion model have largely focused on mutant backgrounds that are susceptible to the GABA_A_ receptor antagonist, pentylenetetrazole (PTZ), a chemical proconvulsant commonly used to induce acute seizures in mammals [2, 18, 19]. While PTZ disruption of GABAergic signaling would be likely to decrease convulsion threshold by altering the excitatory and inhibitory input ratio to muscles, PTZ treatment does not noticeably alter the behavior of wild-type *C*. *elegans* [19]. Convulsion assays that have been used to assess sensitivity to PTZ typically depend on quantifying the cessation of movement of the pharyngeal pumping muscle, a muscle controlled by a mapped neuronal circuit, and posture. However, detecting the effects of PTZ requires convulsion-sensitive animals, such as *lis-1*, *unc-49*, *unc-47*, or *unc-25* mutants [19, 20]. When these mutants are exposed to PTZ, pharyngeal pumping stops and a majority of worms become paralyzed in a dose dependent manner.

Here, we developed an electroshock assay in which we quantitatively monitor paralysis duration and convulsions in *C*. *elegans* following electric shock. This approach has similarities to convulsion models previously established for fruit flies [21–23]. In mammals, the maximal electroshock seizure test, or MEST, is a gold standard to test for anticonvulsant drug activity [24, 25]. We have developed a similar method of inducing convulsions via electric shock in an invertebrate nematode system. Our results indicate that immediately following a brief three-second electric shock, young adult worms exhibit paralysis with body stiffness and elongation. Animals promptly recover from paralysis within seconds after removal of the electric stimulus. The shock impairs normal locomotion and induces a seizure-like behavioral response. Electroshock is a common method of seizure induction used in fly and rodent models; our approach now provides a similar model for the worm.

The widespread use of PTZ in other models of seizure prompted us to investigate the effects of PTZ in our worm electroshock model [26, 27]. PTZ significantly slowed recovery following electric shock. Similarly, *unc-25* and *unc-49* mutants, which lack inhibitory GABAergic neurotransmission, had delayed recovery following shock. To complement these findings, we tested three anticonvulsant drugs commonly used as antiepileptic therapy in humans, retigabine (RTG), sodium valproate (VPA), and levetiracetam (LEV) [28–30]. Since *C*. *elegans* lack voltage-gated sodium channels, we did not select AEDs with Na_v_ as their primary target. In all cases tested, these anticonvulsant compounds improve recovery following electric shock. Interestingly, previous researchers treated *C*. *elegans* with VPA and observed extended lifespan, but the effects of these compounds have not been tested on behavior, or in a worm seizure model [31]. A large-scale RNAi screen using the acetylcholinesterase inhibitor, aldicarb, identified several genes associated with epilepsy in humans that alter inhibitory GABAergic motor neuron function in worms [7]. However, how these genes affect a *C*. *elegans* seizure model has not been evaluated.

Here we have demonstrated that *C*. *elegans* are susceptible to an electric shock method of inducing paralysis and convulsions, similar to well-established methods in other model systems [21–23, 26]. We have also shown that GABAergic neurotransmission plays a role in the time to recovery after electric shock, and that recovery time can be decreased by treatment with antiepileptics and increased with PTZ. The rapid and reliable approach that we have developed could be useful in assessing genetic and pharmacological effects on seizure in worms.

## Materials and Methods

### Animals

*C*. *elegans* were maintained on standard NGM agar plates seeded with OP50 *E*. *coli*. L4 worms were picked and transferred the evening prior to testing and maintained overnight at 25°C. *C*. *elegans* used in these experiments were Bristol N2 strain, CB156 *unc-25 (e156)*, CB382 *unc-49 (e382)*. *C*. *elegans* strains were ordered from the Caenorhabditis Genetics Center (NIH Office of Research Infrastructure Programs, P40 OD010440).

### *C*. *elegans* electroshock assay

**Fig 1:**
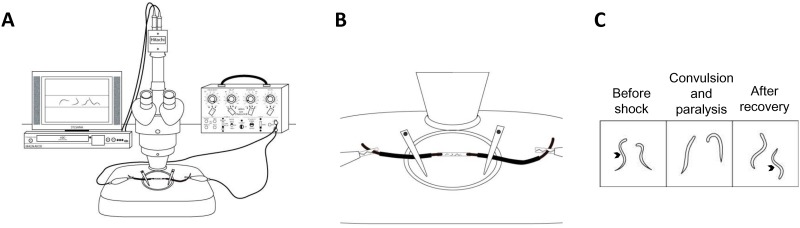
Experimental set up for application of electrical shock in worms. (A) Experimental set up includes a dissecting microscope with an ocular camera. The live image feed is displayed on the TV and recorded with a digital video recorder. (B) A zoomed in view of (A) shows a close up schematic of the experimental tube containing liquid solution and worms with copper wire on either side of tube along with measurements. (C) Schematic of worms before the shock and after recovery are represented with sinusoidal wave body shape (denoted by arrows). During the convulsion and paralysis phase, the worms are generally exhibiting unilateral body bends or paralyzed.

The experimental setup consisted of a Grass SD9 stimulator, Grass SD44 stimulator (used as 3 second timer), dissecting microscope with a camera (Hitachi model KP-D20BU), a twelve-inch television monitor, and an HDD and DVR recorder (Magnavox model MDR535H/F7). A schematic of the setup is shown in Fig 1A and 1B.

On the day prior to experimentation, L4 stage *C*. *elegans* were selected and placed on an NGM agar plate with *OP50 E*. *coli* and stored at 25°C. The tubing utilized was Tygon^®^ microbore tubing with an inner diameter of 0.040”, outer diameter of 0.070”, and wall thickness of 0.015” (Taylor Scientific product number 13-9124-20). The tube was cut into 9mm segments and filled with 15μL of M9 solution or M9 + drug treatment. Thirty minutes prior to stimulation (Grass SD9 stimulator), approximately ten 1-day old adult *C*. *elegans* were picked directly off an NGM plate and transferred to the Tygon tube using a platinum wire pick. (Fig 1B). For pharmacological treatments, drug of interest was dissolved directly into M9 and worms were incubated for a total of 30 minutes. Following incubation, two 4” single gauge insulated copper wires with 1mm diameter were inserted approximately 2mm into either end of the plastic tube. Two alligator clips were attach the two copper wires and connected to a square-pulse generating stimulator (Grass SD9). The copper wires are measured to 1cm apart inside the tube. It is critical that the distance between copper electrodes remain uniform in order to maintain a constant electric field. A shock was delivered for 3 seconds (200 Hz, 3.5ms, 47V) and a microscope camera recorded the shock and subsequent ten minutes. The voltage was selected based on the results of a voltage-response curve using wild-type worms. Voltages between 20V-70V were tested. Recovery was not observed above 60V (S1 Fig). We selected a voltage that correlated with an average recovery time between 40%-50% of the maximum recovery time, which was 47V. Experimental tubes were discarded after stimulation.

After several hours of data were recorded (20–40 individual experiments), the experiments were visually analyzed. The time from the beginning of the stimulus to the time when each individual animal resumed a sinusoidal wave-like swimming motion was recorded. It should be noted that speed of the sinusoidal wave was not taken into consideration when considering recovery, only the wave motion itself. As a result of electrolysis, peripheral bubbles formed on either end of the stimulation tube. Additionally, as seen in S1 Video, *C*. *elegans* occluded by the peripheral bubbles were excluded from analysis. We also noted that a small number of animals did not recover and, therefore, were not considered in recovery analysis. Previous work in *C*. *elegans* has used high temperature to induce seizures [32]. We examined the temperature change of the M9 solution during our stimulus protocol using a high-speed IR imaging system (FLIR) and found temperature only increased by 1±0.5°F during the stimulation.

### Pharmacological manipulations

Drugs were dissolved directly into M9 solution and approximately 15μL of solution was aliquoted into the clear plastic tubing. Drugs of interest were dissolved directly into M9 and worms were incubated for a total of 30 minutes prior to electric shock. The drugs tested were PTZ, RTG, VPA, and LEV, which were obtained from Sigma-Aldrich, St. Louis, MO, USA.

### Statistics

Data was analyzed using One-Way ANOVA followed by a *post-hoc* Multiple Comparisons test (HS = Holm-Sidak) and Student’s *t*-test using SigmaPlot 11.0 (San Jose, California). All bar graphs represent mean ± SEM and asterisks denote significance between bars with all P≤ 0.05, *≤0.05, ***≤0.001, and ns = not significant. “n” is defined as recovery time for one animal where n≥10 and the minimum number of experimental trials per treatment was six. All relevant materials are available by request without restriction.

## Results

### *C*. *elegans* behavioral response to electric shock

**Fig 2:**
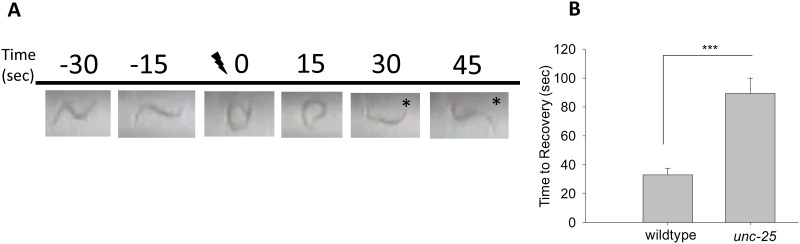
Electroshock results in paralysis and recovery of *C*. *elegans*. (A) Freeze-frame image of a wild-type *C*. *elegans* inside an experimental tube. The images are still frames from a video of the raw data seen in S1 Video and are taken before, during, and after the electric shock (47V, 3 sec). Before the shock, the animal moves in a sinusoidal wave pattern. After the shock, the animal recovers locomotion within ~30 seconds. The recovered worm is represented with an asterisk. (B) Quantification of the visual in (A) depicts the mean recovery time of wild-type (33.0±4.5 seconds), which are compared to *unc-25* mutants (89.5±10.5 seconds). Error bars represent standard error of the mean and significance was determined using Student’s *t*-test where ***P≤0.001 and n≥35.

We set out to develop a model of seizure in *C*. *elegans* using electric shock to induce convulsions similar to practices in other systems [24, 25]. To begin, worms were placed in a transparent plastic tube containing M9 saline solution (Fig 1). Both ends of the tube were plugged with copper wire and connected to a square-pulse generating stimulator. The voltage was chosen based on a voltage-response curve with wild-type worms where average recovery time at 47V is approximately half that of the recovery time at 60V (maximum voltage with recovery; S1 Fig). This method allowed us to assess approximately ten worms per experiment by recording animals with a camera. During a brief electric shock application for 3 seconds, worms display paralysis and elongation (S1 Video, Fig 2A). This is immediately followed by slow unilateral body-bends and convulsions. We define convulsions as repeated unilateral body bends with muscle twitching. With removal of electric shock, convulsions were followed by rapid recovery in which most animals resume sinusoidal, swimming movement (S1 Video). For all experiments, animals were habituated in M9 solution for 30 minutes prior to electric shock.

Shown in Fig 2A is an example of a still image of a wild-type worm visualized before (-30 sec, -10 sec), during (0 sec), and after the electric shock (15 sec, 30 sec, 45 sec). Asterisks highlight time frames in which the worm has recovered from electric shock. The real-time video of the still images in Fig 2A can be viewed in S1 Video. These results indicate that electric shock induces paralysis and convulsions in *C*. *elegans*.

### Paralysis induced by electric shock is sensitive to levels of GABAergic neurotransmission and antiepileptic drugs

**Fig 3:**
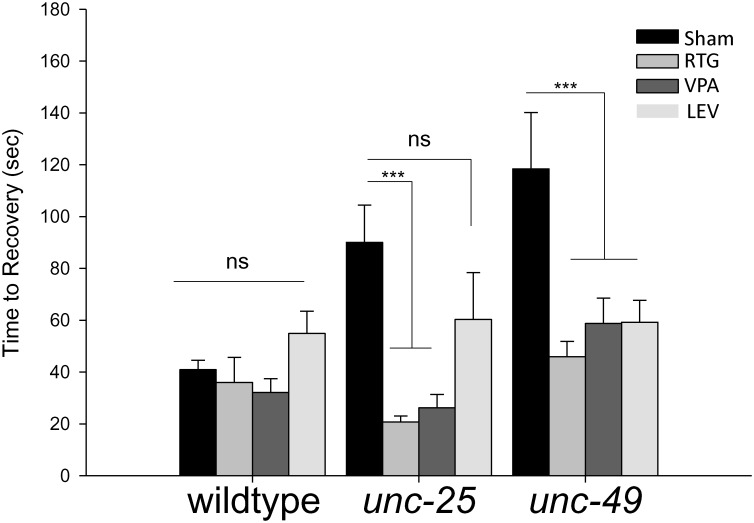
Electroshock recovery time is increased with loss of GABAergic transmission, and improved by antiepileptic drugs. In control conditions, wild-type *C*. *elegans* take significantly less time to recover from an electric shock when compared to loss-of-function mutations in the GABA biosynthetic enzyme *unc-25* or the GABA receptor *unc-49*. Treatments with 3.0 mM LEV, 1.0 mM RTG and 3.0 mM VPA reduce *unc-25* and *unc-49* mutant recovery. Error bars represent standard error of the mean and significance was determined using two-way ANOVA and Holm-Sidak multiple comparisons test where ***P≤0.001. ns = not significant P>0.05 and n≥17.

*C*. *elegans* moves by generating repeated sinusoidal body bends in a given direction. While somewhat of an oversimplification, generally body bends are generated by excitatory cholinergic motor neurons stimulating contraction of body wall muscles on one side of the animal, while inhibitory GABAergic motor neurons trigger relaxation of body wall muscles on the opposing side of the animal [8, 9, 11, 33–37]. To determine how changes in motor neuron function affect recovery time following electric shock, we tested *unc-25* mutants, which are unable to synthesize GABA. A strong loss-of-function allele of *unc-25*, *e156* [8], slowed recovery significantly compared to wild-type animals as shown by S2 Video and quantification in Fig 2B. Quantification of recovery time demonstrated that wild-type animals recover in 33.0±4.5 seconds compared to *unc-25* mutants which recover in 89.5±105 seconds (Student’s *t*-test, T_(2,73)_ = -4.681, P<0.001, Fig 2B). Loss of function in *unc-49*, the GABA_A_ receptor on muscles, also delayed recovery (Fig 3). These results indicate that loss of inhibitory GABAergic transmission at the neuromuscular junction leads to increased sensitivity to electric shock.

To further validate our electroshock assay as a model of seizure, we tested how three FDA approved antiepileptic drugs, LEV, RTG and VPA, affect recovery following electric shock. The effects of LEV and RTG have not been tested in *C*. *elegans* to our knowledge. VPA has been used previously to investigate dopaminergic neurodegeneration, but has not been assessed in a *C*. *elegans* model of seizure [38]. While none of these drugs affected outcomes in wild-type animals (Fig 3), the delayed recovery of *unc-25* mutants was strongly suppressed with RTG and VPA treatment, and trended towards suppression with LEV treatment (Fig 3). Similarly, treatment of *unc-49* mutants with all three antiepileptic drugs resulted in suppression of slow recovery following shock (Fig 3). Collectively, these results show that our electroshock assay is highly sensitive to mutations that reduce inhibitory GABAergic transmission to muscles, an effect that can be improved by application of multiple antiepileptic drugs.

### PTZ increases time to recovery

**Fig 4:**
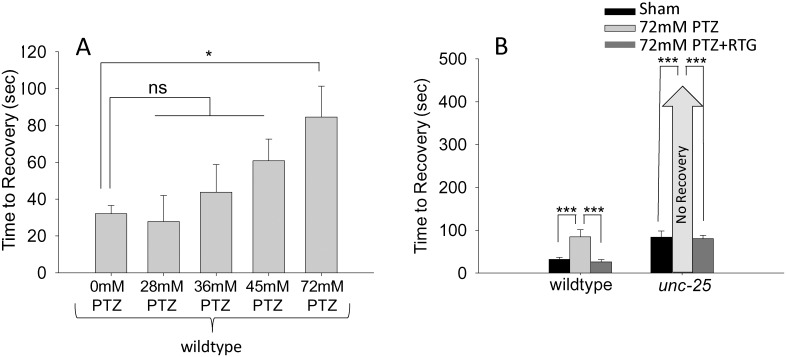
The GABA receptor antagonist PTZ increases recovery time following electroshock. (A) Electric shock recovery times for wild-type animals treated with varying doses of PTZ. (B) 72 mM PTZ extended recovery time of wild-type *C*. *elegans* and enhanced deficits in recovery of *unc-25* mutants. Both genotypes showed improved recovery when exposed to a cocktail of 72 mM PTZ + 1 mM RTG. Error bars represent standard error of the mean and significance was determined using Student’s *t*-test where ***P≤0.001 and *P≤0.05 ns = not significant and n≥15.

**Fig 5:**
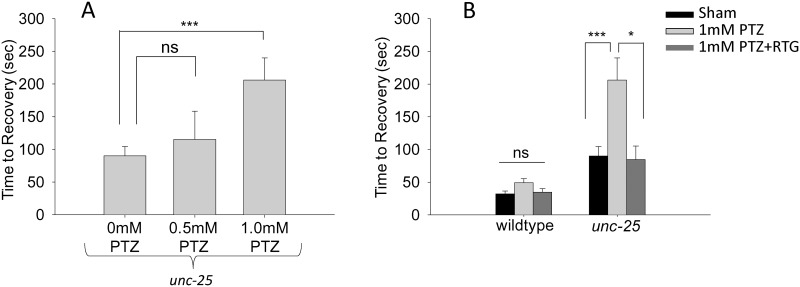
Low concentrations of PTZ increase recovery times in *unc-25* mutants. (A) Electric shock recovery times for *unc-25* mutants treated with varying doses of PTZ. (B) 1 mM PTZ significantly extended recovery time of *unc-25* mutants. This effect was reversed by treatment of *unc-25* mutants with a cocktail of 1 mM PTZ + 1 mM RTG. Error bars represent standard error of the mean and significance was determined using Student’s *t*-test where ***P≤0.001 and *P≤0.05 ns = not significant P>0.05 and n≥13.

To pharmacologically assess how altering GABAergic transmission affects recovery from electric shock, we treated wild-type animals with PTZ. In wild-type animals, a dose response curve suggested a concentration of 72mM was enough to significantly slow recovery compared to sham controls (Student’s *t-*test, T_(2, 63)_ = -2.35, P≤0.05, Fig 4A). To our knowledge, this is the first example of a behavioral assay in which wild-type *C*. *elegans* are susceptible to PTZ, as previous studies did not detect responses in wild-type animals to even PTZ concentrations as high as 145mM (approximately 20mg/mL) [19]. The effect of PTZ we observed was suppressed by co-treating animals with RTG, consistent with PTZ inhibiting the GABA receptor and GABAergic function (Fig 4A). Unexpectedly, when *unc-25* mutants were exposed to 72mM PTZ they did not recover suggesting survival might be lost in these animals after electric shock (>400 seconds, Fig 4B). However, when *unc-25* animals were exposed to a cocktail of 72mM PTZ and 1mM RTG, the worms showed drastically improved recovery following electroshock (80.4±7.8 seconds, Fig 4B). *unc-25* animals were then exposed to concentrations of PTZ that were significantly less than 72mM to determine if *unc-25* would recover at lower concentrations of PTZ (Fig 5A). A dose response curve for PTZ determined *unc-25* recovered locomotion after 30 minutes of exposure followed by electric shock and that 1mM PTZ exposure took significantly longer to recover compared to control (Student’s *t*-test, T_(2, 44)_ = -3.324, P<0.05). Similar to results with higher concentration of PTZ, a cocktail of 1 mM PTZ and 1mM RTG significantly decreased time to recovery (Student’s *t-*test, T_(2, 34)_ = 2.534, P<0.05). These pharmacological results complement our genetic experiments, and re-enforce the concept that reducing GABAergic motor neuron function increases susceptibility to electroshock.

## Discussion

In this study, we have designed, developed and validated an electroshock assay to investigate seizure-like behavior in *C*. *elegans* (Figs 1 and 2). We have termed the behavioral response following electroshock as seizure-like based on the following observations. 1) Following electroshock of wild-type animals, we observed paralysis that was followed by physical seizing of the worm in which hypercontraction of muscles and unilateral, extremely slow body bends occur (S1 Video). This is reminiscent of seizing that occurs in response to electroshock in other models of seizure [19, 23, 25]. 2) The rescue of electroshock phenotypes by multiple AEDs (Fig 3). 3) The interruption of GABA transmission causing increased susceptibility to electroshock, which we tested with both genetic intervention using *unc-25* and *unc-49* loss of function (lf) (Fig 3), and pharmacological intervention using PTZ (Figs 4 and 5). 4) Finally, electroshock is an established model for inducing seizure in both rodents and flies [21–25]. These observations and lines of reasoning collectively argue that our electroshock model in worms is likely to be a valid model of seizure for this system.

Treating worms with several antiepileptic drugs approved for human use improved defects in recovery from electroshock caused by genetically or pharmacologically impairing GABAergic transmission (Figs 4 and 5). In *unc-25* and *unc-49* mutants, loss of inhibitory, GABAergic transmission to muscles significantly slowed recovery to normal sinusoidal body motion following electroshock compared to wild-type animals (Figs 2B and 3). This is significantly improved by treating worms with RTG, VPA and LEV. Because GABAergic transmission inhibits muscle contraction, *unc-25* and *unc-49* are classic hyperexcitability mutants, which have behavioral and neuronal abnormalities that are well characterized [8, 9, 11, 39]. A widely used behavioral assay that analyzes paralytic activity is the aldicarb assay [40]. Aldicarb is an acetylcholinesterase inhibitor that causes accumulation of acetylcholine at the neuromuscular junction, which in turn induces paralysis over time due to hyperexcitation of muscles. RNAi screens have identified many mutants with GABA transmission defects, including *unc-25* and *unc-49*, which are hypersensitive to aldicarb and paralyze more rapidly than wild-type animals [7]. While the aldicarb response is different than our electroshock assay, our results show that the underlying mechanisms that affect sensitivity to electroshock and aldicarb are similar. This suggests that our electroshock assay is likely to be a rapid and quantitative behavioral readout for the ratio of excitatory to inhibitory input onto muscles.

Why do the antiepileptic drugs RTG, VPA and LEV rescue the increased recovery time that *unc-25* and *unc-49* mutants display following electroshock? Our AEDs were specifically chosen because they do not target Na_v_ channels, as *C*. *elegans* lacks these channels. Prior work suggests that LEV may target the synaptic vesicle protein SV2A in mammals [41, 42]. The target of LEV is unknown in *C*. *elegans*, and there is no direct ortholog of SV2A in worms. However, a distant homolog of SV2A, called SVOP, is orthologous to SVOP-1 in *C*. *elegans*, and could be a potential LEV target [43].

RTG and its targets are well studied in mammals, but RTG has not been investigated before in worms. In mice, RTG activates the neuronal voltage-gated potassium channels KCNQ 2/3 [44–46]. *C*. *elegans* has three KCNQ-like channels KQT-1, KQT-2, and KQT-3. Based on the mechanism of action in mice, and the presence of conserved molecular targets in worms, it is most likely that RTG reduces excitation in the muscles or the cholinergic motor neurons by blocking one or more of these KQT channels. As a result, RTG would compensate for the reduced inhibitory GABA transmission in *unc-25* and *unc-49* mutants thereby rescuing increased electroshock sensitivity in these animals [46–48]. Work in rat hippocampal slices has suggested that RTG at high concentrations can increase GABA synthesis [49]. While this mechanism of action seems unlikely, as *unc-25* is reportedly a null and *unc-49* mutants would not be sensitive to changes in GABA synthesis, there is evidence that there could be very low concentrations of GABA in *unc-25* mutants suggesting increased GABA synthesis might be possible in this animal [50]. In worms, VPA is thought to inhibit acetylcholine release and possibly ERK-MAPK signaling [38]. Given that increased sensitivity of *unc-25* and *unc-49* mutants to electroshock is due to a loss of inhibitory GABAergic transmission, and an ensuing excess of cholinergic excitation, it is reasonable that blocking acetylcholine release with VPA would rescue electroshock defects in these animals. The response of worms to antiepileptic drugs with efficacy in humans is important, as it suggests our assay could potentially be used as a screening tool for novel compounds that could affect neuronal excitability in humans. While all the reagents used in this study are water soluble, we included a concentration curve for dimethyl sulfoxide (DMSO) which shows there is no significant difference in recovery from electric shock in concentrations up to 0.5% DMSO (S2 Fig). This is important because of the potential for this assay to be used as a high-throughput drug screen, which may require DMSO as a solvent.

The effects of PTZ, a GABA receptor antagonist, on wild-type animals provides further pharmacological evidence that reduced GABAergic transmission affects responses to electroshock. Our results demonstrate that wild-type animals respond to PTZ in a dose dependent manner. The concentrations of PTZ selected for analysis of wild-type animals were based on prior studies; however, in these studies, PTZ was mixed into the NGM agar plates whereas our protocol fully submerges the worms in PTZ dissolved in M9. Exposure on plates could alter vulnerability to PTZ, which could explain why wild-type worms were not susceptible to PTZ in previous plate-based assays, but are susceptible to PTZ in our electroshock assay. Alternatively, our electroshock assay might be particularly sensitive to the ratio of excitatory to inhibitory input to muscles making it highly sensitive to PTZ. Doses of PTZ used in combination with RTG were selected based on worms being able to recover from the convulsions, but taking longer than sham treated animals. For wild-type worms, 72mM PTZ was used whereas 1mM was selected for *unc-25* mutants, since *unc-25* did not survive a 30-minute habituating treatment with 72mM PTZ. This enhancer effect of PTZ with *unc-25* (lf) was somewhat surprising. We tested a strong loss-of-function allele, *e156*, for the only known GABA synthesis gene in *C*. *elegans*, *unc-25*. *unc-25* (lf) showed a large enhancer effect when exposed to several different concentrations of PTZ. Previous studies suggest that *e156* is potentially a complete null for *unc-25* and GABA synthesis [8, 9, 19]. If this were the case, one would predict that *unc-25 (e156)* mutants should not have increased behavioral responses to PTZ. However, our experiments demonstrate that delayed recovery of *unc-25* mutants following electroshock is dramatically enhanced with PTZ treatment. There are several potential explanations for this result. First, there is evidence that PTZ might block not only GABA receptors, but also reduce calcium channel selectivity and depolarize the cell membrane [51]. Second, it was previously suggested that *unc-25* may not be a null, but a strong hypomorph for GAD, in which case very low, but physiologically critical, concentrations of GABA would be present but not detected by immunohistochemistry [50]. In this case, enhancer effects could result from the complete abolishment of GABAergic transmission to muscles. Given the much milder effects of PTZ on recovery of wild-type animals following electroshock, it is possible that off target effects of PTZ are only observed once GABAergic transmission is completely or largely removed. Finally, the enhancer effect of PTZ with *unc-25* (lf) could result from both *unc-25* being a hypomorph, and PTZ having effects on GABA receptors as well as calcium channels.

Electroshock is one of the most common models of acute and chronic seizure in mammals. We have now developed a similar electroshock assay for *C*. *elegans*. Because it is inexpensive, rapid and has shown relevance with existing antiepileptic drugs, our *C*. *elegans* electroshock assay has the potential to become an initial screening tool for human seizure therapeutics. Further, our assay could act as a complement to other approaches that alter cellular excitability in worms, such as aldicarb sensitivity [7]. Thus, the electroshock assay we have developed for *C*. *elegans* has the potential to provide molecular and cellular insights that are complementary to other whole organism systems, such as flies and rodents. Additionally, since worms lack voltage gated sodium channels, our electroshock assay is ideally suited for identifying novel AEDs that do not target sodium channels. This characteristic, along with the low cost and high throughput potential of our assay, could provide significant benefits for AED drug discovery over other more established models of seizure.

Future experiments will be needed to further validate our electroshock assay as a model of seizure. For example, optogenetic and further pharmacological manipulation of the excitatory and inhibitory inputs to muscles could be analyzed for impacts on electroshock recovery. This could provide further support for our proposed model that the response to electroshock is impacted by excitatory to inhibitory transmission balance on muscles. Further, genes and pathways that affect fly seizure models might be tested in order to understand the relevance of our electroshock assay to other existing seizure models [21–23, 52].

## Supporting Information

S1 Fig A voltage response curve shows the recovery time after electric shock of wild-type worms between 20V-60V. (TIF)

S2 Fig A DMSO sham concentration curve at 0.1% and 0.5%, and demonstrates these concentrations have no significant effect on wild-type recovery time after electric shock. (TIF)

S1 Video This video shows images of the experimental setup and annotated footage of actual raw data. Examples of the experimental setup, followed by raw data video of wild-type worms in control conditions. There are five worms in this experimental tube and recovery times for all five are recorded.
(MP4)

S2 Video *unc-25* mutant video of before and after electroshock. Mutant worms deficient in GABA display slightly altered locomotion patters compared to wild-type. In this raw data example of *unc-25*(e156) in control conditions, the middle five worms are considered for analysis since the worm on the far right of the video is not clearly visible. Of the five worms, three do not completely recovery; therefore, only two recovery times are recorded from this video.
(MP4)
