# Supplementary figures and images for: Modulating Behavior in C. elegans Using Electroshock and Antiepileptic Drugs
Source: PLoS One. 2016 Sep 26;11(9):e0163786. doi: 10.1371/journal.pone.0163786 (PMC5036823; doi:10.1371/journal.pone.0163786)

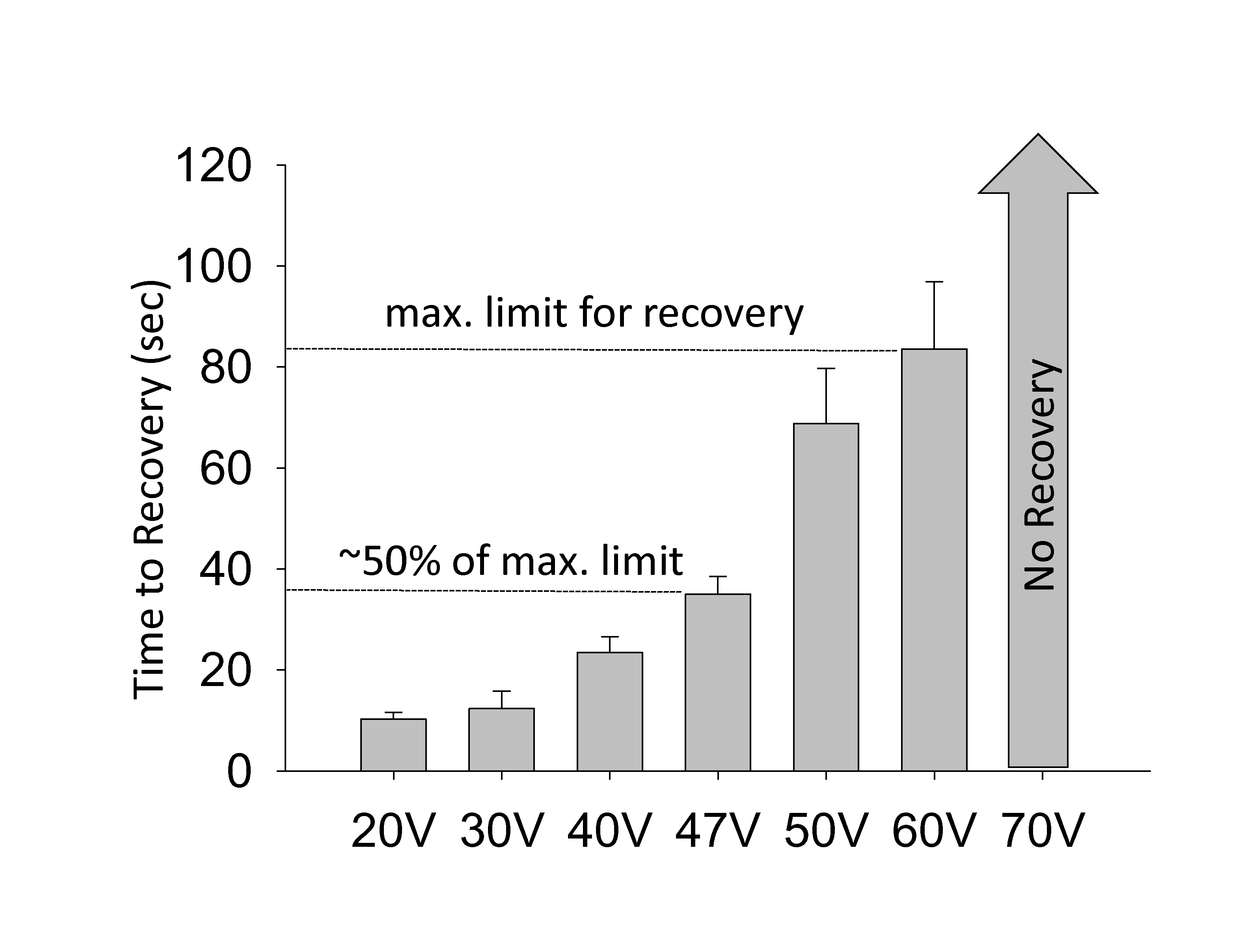

Supplement: S1 Fig — (TIF) [file pone.0163786.s001.tif]

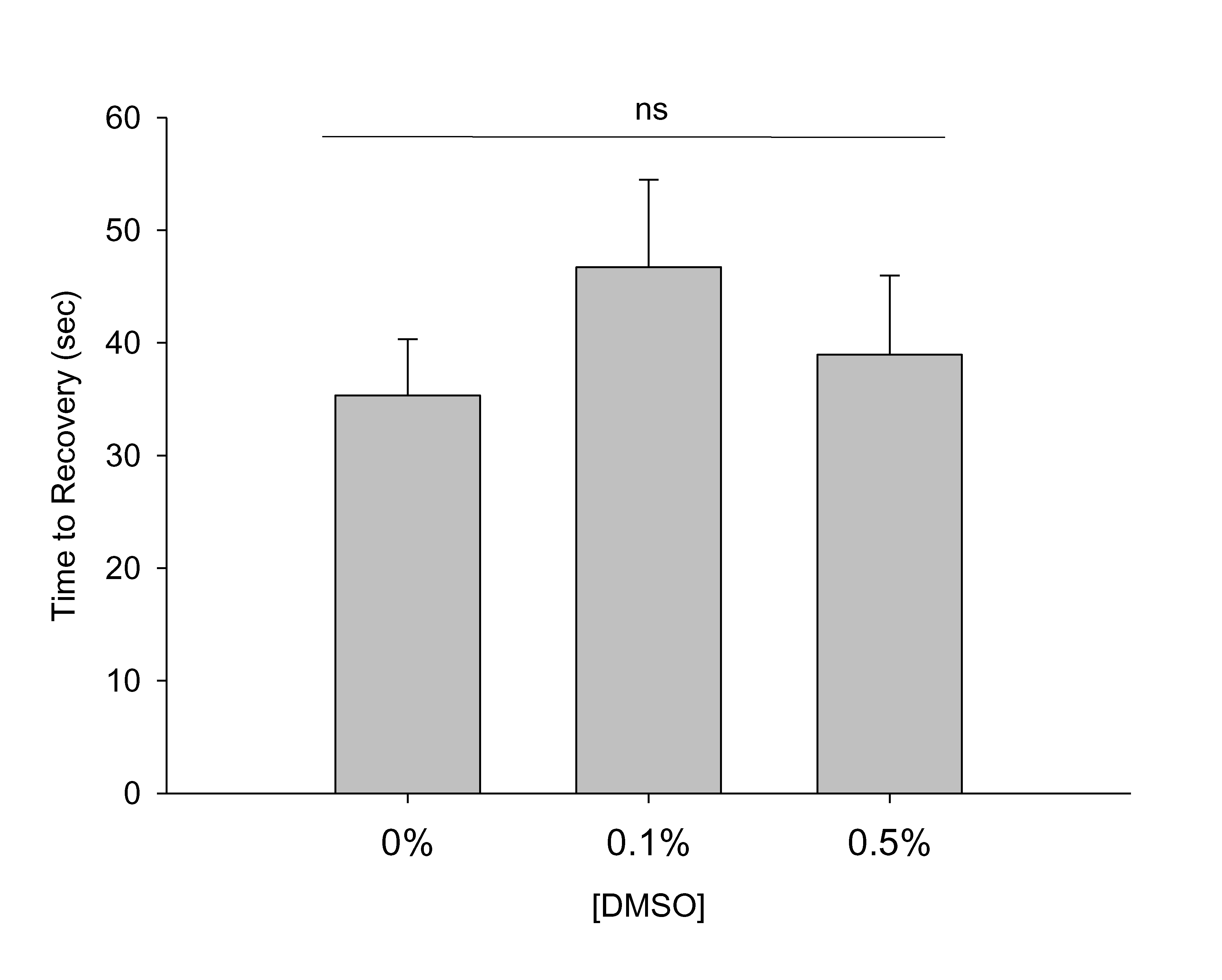

Supplement: S2 Fig — (TIF) [file pone.0163786.s002.tif]
